# When to Operate in Cleft Palate

**Published:** 1903-10-03

**Authors:** 


### WHEN TO OPERATE IN CLEFT PALATE.

The question of the best time to operate for cleft palate is a matter on which there is still a pretty wide divergence of opinion among surgeons. Dr. Brophy,[1] of Chicago, takes the view that the most desirable time of life to close the cleft is within five months of birth, and some of his most satisfactory results have been obtained in infants from ten days to three weeks old. He points out that in an infant who has a cleft palate the upper jaw is usually broader than the lower jaw. Moreover, the difference in width of the upper, as compared with the lower jaw, is the difference between the edges of the cleft, and so the theory that has long been accepted that the cleft palate is the result of incomplete development of the tissues which enter into its formation is erroneous. There is abundance of tissue to form a perfect palate, but, unfortunately, during the development of the child, the edges of the bone fail to unite. This being the case the lower jaw, as soon as the muscles of mastication become active, bring pressure to bear upon the upper jaw and hard palate, and acting as a wedge tend to force the two halves of the bone further apart. The object of the surgeon should be, therefore, Dr. Brophy argues, to bring the two maxillary bones together bodily, and not merely to close the cleft. By bringing the two halves of the upper jaw into apposition, not only is the cleft in the palate closed, but the shape of the nasal cavities is restored to the normal, and the upper molars are made to coapt with their fellows in the lower jaw. The operation, Dr. Brophy has found, can be satisfactorily performed during the first five months of life, when the bones are soft and easily bent. Briefly stated the procedure he describes is as follows:—the edges of the cleft are pared, the two halves of the upper jaw are approximated by external pressure and fastened together by wire sutures passed through the edges of the hard palate. Among the reasons why he believes early operation to be desirable, Dr. Brophy mentions the following in addition to the one already given, namely, the fact that the bones are soft and easily bent in the first few months of life: (1) Surgical shock is less because the nervous system of a young child is not well developed. Hæmorrhage is slight, and all mental impressions are eliminated; (2) if the muscles are brought into action early they develop instead of undergoing atrophy, and hence a good velum is secured with plenty of tissue, whereas if operation be undertaken later the parts are so shrunken from disuse that they never properly recover; (3) the alveolar margin of the upper jaw is brought into its proper relation with that of the lower jaw, and the nasal cavities are restored to their proper form; (4) when the child learns to talk no nasal accent is developed. If the operation is not done until faulty habits of speech have been acquired it is with difficulty that they can be overcome. In cases where the cleft palate is associated with hare-lip, Dr. Brophy prefers to attack the palate first, as the surgeon then has more room for the necessary manipulation.

[1] Brooklyn Med. Jour., Sept., 1903.

